# Feasibility Study on Inflammasome Proteins as Biomarkers in the Cerebrospinal Fluid of Pediatric Patients with Hydrocephalus Due to Intraventricular Hemorrhage

**DOI:** 10.3390/biom16010035

**Published:** 2025-12-25

**Authors:** MaryLourdes Andreu, Alexandra Castellanos, Robert W. Keane, Juan Pablo de Rivero Vaccari, Jennifer C. Muñoz Pareja, Helen M. Bramlett, W. Dalton Dietrich, Nadine A. Kerr, Heather J. McCrea

**Affiliations:** 1 Miami Project to Cure Paralysis, University of Miami Miller School of Medicine, Miami, FL 33136, USA; mxa1485@med.miami.edu (M.A.); rkeane@med.miami.edu (R.W.K.); jderivero@med.miami.edu (J.P.d.R.V.); jcm457@med.miami.edu (J.C.M.P.); hbramlett@med.miami.edu (H.M.B.); ddietrich@med.miami.edu (W.D.D.); 2 Medical Scientist Training Program, University of Miami Miller School of Medicine, Miami, FL 33136, USA; 3 Department of Neurological Surgery, Jackson Memorial Hospital Miami, University of Miami Miller School of Medicine, Miami, FL 33136, USA; axc3009@med.miami.edu; 4 Department of Cellular Physiology and Molecular Biophysics, University of Miami Miller School of Medicine, Miami, FL 33136, USA; 5 Division of Pediatric Critical Care, Department of Pediatrics, Jackson Memorial Hospital, University of Miami Miller School of Medicine, Miami, FL 33136, USA; 6 Bruce W. Carter Department of Veterans Affairs Medical Center, Miami, FL 33125, USA; 7 Department of Pediatrics, Jackson Memorial Hospital, University of Miami Miller School of Medicine, Miami, FL 33136, USA

**Keywords:** pediatric hydrocephalus, intraventricular hemorrhage (IVH), inflammasome

## Abstract

Pediatric hydrocephalus results from the overproduction, obstruction, or inability to resorb cerebrospinal fluid (CSF), which may lead to ventricular enlargement. Hydrocephalus may be secondary to other pathologies, including intraventricular hemorrhage (IVH). Surgical intervention may include a ventricular access device or a ventriculosubgaleal shunt in premature infants who are too small for permanent CSF diversion. Hydrocephalus, an inflammatory pathology, involves activation of the inflammasome. Elevation in pro-inflammatory proteins is often associated with worsening clinical outcomes in pediatric pathologies. Whether inflammasome proteins are reliable biomarkers for pediatric hydrocephalus has not been established. We analyzed CSF samples via an ELLA assay from pediatric hydrocephalic patients with IVH of prematurity vs. controls and compared changes in inflammasome protein concentrations post-reservoir surgery at different time points. The area under the curve was calculated using receiver operator characteristic curves. The levels of apoptosis-associated speck-like protein containing a caspase recruitment domain (ASC), caspase-1, and interleukin-18 were significantly elevated in pediatric hydrocephalic patients with IVH vs. controls. Thus, these proteins are reliable biomarkers of the inflammatory response in pediatric hydrocephalus following ROC analysis, with an AUC of 1.0 and high sensitivity and specificity at the corresponding cut-off points. Ongoing work seeks to validate these findings in a larger cohort of pediatric hydrocephalic patients with varying etiologies.

## 1. Introduction

Pediatric hydrocephalus is a complex neurological condition caused by varying etiologies. Hydrocephalus involves an increase in cerebrospinal fluid (CSF) levels due to lack of resorption, overproduction, or obstruction from outflow, which leads to ventricular enlargement. A buildup of CSF can lead to rapid head growth and/or ventricular enlargement. This can lead to the need for surgical intervention in very young babies [1,2]. Pediatric hydrocephalus is estimated to have a prevalence between 1 and 32 per 1000 births, with variation depending on the population evaluated [3]. The onset of hydrocephalus may be secondary to other pathologies, including intraventricular hemorrhage (IVH) [4]. The prevalence of any grade of IVH in premature neonates is approximately 34.3% [5]. IVH in the preterm infant involves the rupturing of fragile blood vessels leading to bleeding in or around the brain’s ventricles and may increase the likelihood of brain injury and long-term neurological deficits [6,7,8,9]. It is a multifactorial condition that may be precipitated by the fragility of the germinal matrix vasculature, increased cerebral pressure, decreased cerebral blood flow, and coagulation disturbances [6,10,11,12,13,14,15].

Hydrocephalus requires timely surgical intervention to improve clinical prognosis [16,17]. In infants who are too small for permanent CSF diversion, surgical intervention may include a ventricular access device (VAD) or a ventriculosubgaleal shunt (VSGS) [18]. These temporizing procedures allow for CSF drainage until the child is old enough for a permanent CSF diversion procedure such as a ventriculoperitoneal shunt (VP shunt) or endoscopic third ventriculostomy (ETV). VAD involves the insertion of a ventricular catheter attached to a reservoir in the subgaleal space, which is placed in the frontal region at the junction of the fontanelle and coronal suture [19]. The medical team is subsequently able to perform serial CSF percutaneous taps to remove fluid, which is typically discarded [19]. This allows access to CSF at varying time points after the initial event that caused hydrocephalus. A reservoir is typically not a permanent treatment because it requires repeated access by the clinician. In a limited number of cases, hydrocephalus resolves during the treatment with the temporizing procedure, but in most of these patients, it does not. Those who continue to have hydrocephalus will require a VP shunt or ETV to permanently treat their hydrocephalus when they are older [20,21].

Hydrocephalus is considered a pro-inflammatory disease with activation of both the innate and adaptive immune responses [11,19,22,23]. In pro-inflammatory pathologies, such as traumatic brain injury (TBI), Alzheimer’s disease, and subarachnoid hemorrhage, there is an elevation of serum inflammasome pathway proteins [24,25,26,27,28]. Clinicians and researchers have investigated emerging genomic and brain imaging techniques, as well as cell-based therapies, to mitigate the inflammatory cascade [29,30,31]. However, there have been safety concerns for implementation of these therapies [30,32]. Additionally, given the different etiologies of hydrocephalus in pediatric patients and neonates compared to adults, there is a need for further research in these populations to identify whether inflammasome proteins can be used as biomarkers in pediatric hydrocephalus post-surgical intervention [33]. The identification of biomarkers for pediatric hydrocephalus, particularly the inflammasome proteins, apoptosis-associated speck-like protein containing a caspase recruitment domain (ASC), caspase-1, and interleukin-18 (IL-18), could potentially serve as a diagnostic and prognostic tool, as well as help identify a potential subset of infants who may not require permanent treatment. It could also potentially help suggest targets for future therapeutic interventions. Through this feasibility study, we hypothesized that inflammasome proteins in the CSF of pediatric hydrocephalic patients are (i) detectable, (ii) elevated compared to healthy controls, (iii) potential biomarkers. We analyzed CSF samples from five pediatric hydrocephalic patients with IVH of prematurity compared to three healthy controls to assess the changes in inflammasome protein concentrations over time after placement of a reservoir to treat hydrocephalus. We specifically assessed ASC, caspase-1, and IL-18. We also calculated the area under the curve (AUC) using receiver operator characteristic (ROC) to evaluate the potential suitability of these inflammasome proteins as biomarkers for pediatric hydrocephalus.

## 2. Materials and Methods

### 2.1. Participants

The CSF samples for this study were obtained from pediatric patients with hydrocephalus at our institution who were treated with VAD and from non-hydrocephalus pediatric controls whose CSF was purchased commercially. Premature infants with hydrocephalus due to IVH (N = 5, n = 3 males, n = 2 females) were admitted to the Neonatal Intensive Care Unit (NICU) at Jackson Memorial Hospital, Holtz Children’s Hospital. Once a decision was made to place a VAD to treat hydrocephalus, families were approached for participation in this study. Informed consent to participate in this clinical study was obtained from a parent according to the University of Miami Miller School of Medicine/Jackson Memorial Hospital IRB protocol #20210352. Five patients received a VAD (reservoir surgery) for hydrocephalus resulting from IVH of prematurity. CSF samples were obtained via serial taps through the reservoir port, which were obtained daily or at longer intervals based on clinical need for tap, and CSF, which would otherwise have been discarded, was sent to the lab for analysis. Four of the five patients ultimately required permanent CSF diversion via a VP shunt. The control samples (n = 3) were obtained from Medix Biochemica USA, Inc. (Maryland Heights, MO, USA) from pediatric patients (0 years old) who underwent spinal tap due to suspected neurological diseases but tested negative for these pathologies. Prior to shipment, the company stored the samples at −20 °C, and they subsequently shipped them in dry ice to our laboratory.

### 2.2. CSF Collection

After the CSF samples were collected and transferred, the falcon tubes were temporarily placed on ice for transport from the NICU to the lab. If blood was present in the CSF, the samples were centrifuged at 2000× *g* for 10 min at 4 °C. The clear supernatant was then transferred to 2.0 mL conical vials. If no blood was present in the CSF, the samples were transferred to 2.0 mL conical vials. The CSF collection time points (CTPs) post-reservoir included the following ranges: CTP 1: Day 1, CTP 2: Day 4–5, CTP 3: Day 7–8, CTP 4: Day 14–15, CTP 5: Day 18–20, CTP 6: Day 22, CTP 7: Day 28–29, CTP 8: Day 41. CSF samples were collected for clinical, rather than research purposes for each patient. We strove to match the collection days as closely as possible. Additionally, given that IVH patient 5 was not as clinically stable as the other four participants, CSF CTPs were limited for this baby. Patient 5 did not require a shunt. This patient was initially tapped through the reservoir to treat hydrocephalus. The fontanelle subsequently became sunken, and taps were stopped. This baby had severe cortical volume loss and did not require a shunt since taps were weaned.

### 2.3. ELLA Simple Plex Assay

An ELLA system (ProteinSimple, Bio-Techne Corporation, Minneapolis, MN, USA) assay was performed for CSF pediatric patients with hydrocephalus due to IVH and non-hydrocephalus controls following previously published methodology [25,34,35]. Briefly, a 32-well custom ELLA plate was utilized. Thirty-two corresponding conical vials were obtained. Diluent reagent (30 µL/vial) and CSF samples (30 µL/vial) were added to each vial. Subsequently, 50 µL of this mixture was transferred to the corresponding well. The washing buffer (1 mL/washing buffer well) was loaded in the ELLA plate. The ELLA assay ran for 80 min and provided the raw protein concentrations (pg/mL).

### 2.4. Biomarker Statistical Analysis

**Figure 1:**
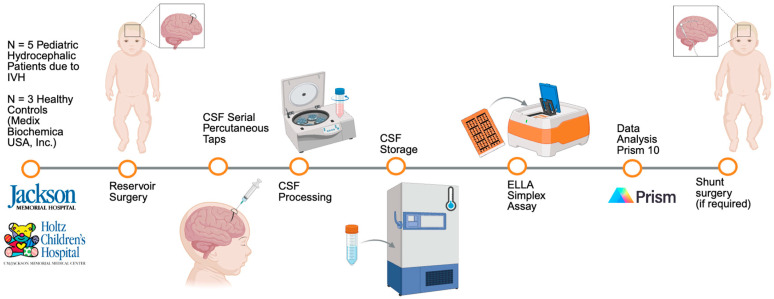
Methodology timeline: n = 5 pediatric hydrocephalic patients due to IVH all received a VAD (reservoir surgery) and underwent serial percutaneous taps. n = 3 samples from Medix Biochemica USA, Inc. served as the healthy controls. IVH CSF samples with blood were centrifuged. All samples were stored in a −80 °C freezer. CSF samples run on ELLA Simplex Assay, and concentration levels (pg/mL) were analyzed using Prism10. If taps could not be weaned, pediatric hydrocephalic patients went on to undergo a permanent shunt once clinically ready. Created in BioRender. Andreu, M. (2025) https://app.biorender.com/illustrations/67c3b905e6be82cc82b2ca23 (accessed on 22 December 2025).

The inflammasome proteins analyzed included the following: (i) ASC, (ii) caspase-1, and (iii) IL-18. The results were graphed using Prism10. For each inflammasome protein, the results of pediatric hydrocephalic patients with IVH (n = 5) were graphed and compared to the averaged controls (n = 3). For comparisons between the patients and controls, a two-way ANOVA, followed by a Šídák’s multiple comparisons post hoc test, was performed for each inflammasome protein graph at each CSF collection time point. For comparisons between the averaged patients and controls, a paired t-test was performed for each inflammasome protein graph at each CSF collection time point. Lastly, to evaluate the suitability of these biomarkers in pediatric hydrocephalus, two ROC analyses were performed. In the first analysis, CTP 1 for ASC, caspase-1, and IL-18 were analyzed, since this was the most acute time-point post-reservoir surgery to detect discriminatory performance between IVH patients and controls. The datasets were then used to generate ROC metrics (cut-off, sensitivity, specificity, AUC) and were subsequently fitted in ROC curves. In the second analysis, for each inflammasome protein, the ROC data were generated separately at each of the eight CTPs. At each CTP, the dataset included five values for the IVH patients versus three values for the healthy controls. Figure 1 provides an overview of the methodology timeline (Figure 1).

## 3. Results

### 3.1. ASC, Caspase-1, and IL-18 Are Detectable in the CSF of Pediatric Patients with Hydrocephalus Due to IVH

**Figure 2:**
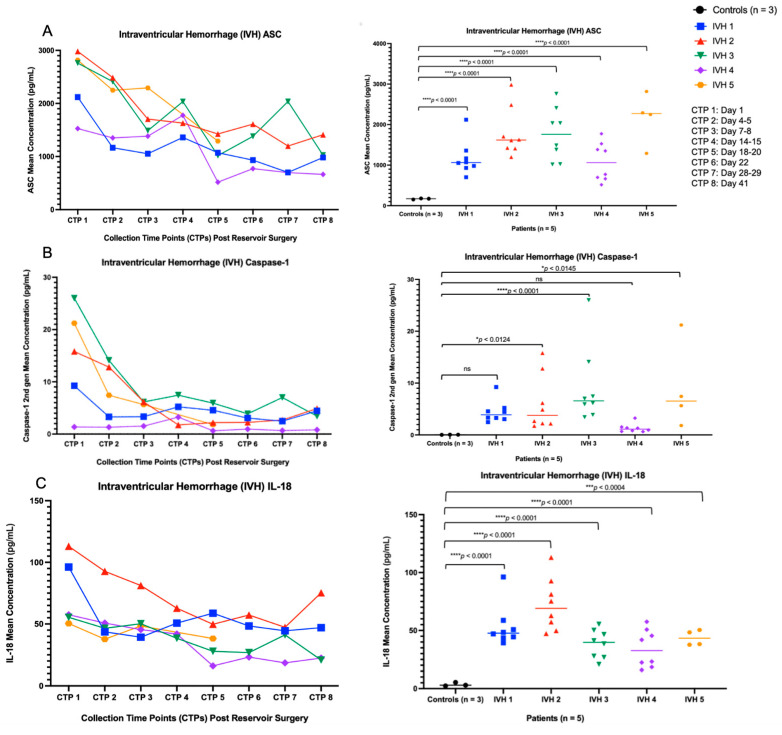
Inflammasome protein concentration levels. (**A**) The two-way ANOVA for ASC was significant, **** *p* < 0.0001, *F*(5, 31) = 28.87, and Šídák’s test was significant between IVH patients 1, 2, 3, 4, and 5 vs. controls (n = 3) **** *p* < 0.0001. (**B**) The two-way ANOVA for caspase-1 was significant, **** *p* < 0.0002, *F*(5, 31) = 7.060, and Šídák’s test was significant between IVH patient 2 vs. controls (n = 3), * *p* < 0.0124; IVH patient 3 vs. controls (n = 3), **** *p* < 0.0001; IVH patient 5 vs. controls (n = 3), * *p* < 0.0145; and not significant (ns) between IVH patient 1 vs. controls (n = 3), *p* < 0.1057, and between IVH patient 4 vs. controls (n = 3), *p* < 0.9658. (**C**) The two-way ANOVA for IL—18 was significant, **** *p* < 0.0001, *F*(5, 31) = 30.15, and Šídák’s test was significant between IVH patients 1, 2, 3, 4 vs. controls (n = 3), **** *p* < 0.0001, and between IVH patient 5 vs. controls (n = 3) *** *p* < 0.0004. Given that the controls (n = 3) each had a single collection time point, these three samples are represented separately from the CTPs.

The participants in our feasibility study consist of premature neonates. The ELLA Simple Plex Assay demonstrated that inflammasome protein concentrations (i.e., ASC, caspase-1, and IL-18) are present in the CSF of these patients (Figure 2).

### 3.2. ASC, Caspase-1, and IL-18 Concentration Levels in the CSF of Pediatric Patients with Hydrocephalus Due to IVH Are Elevated Compared to Healthy Controls

A two-way analysis of variance (ANOVA) was conducted across all time points to compare each inflammasome protein concentration levels (i.e., ASC, caspase-1, IL-18) vs. controls (n = 3). The ASC concentration levels, analyzed using a two-way ANOVA, were significantly higher in the CSF of IVH patients compared with controls, *F*(5, 31) = 28.87, **** *p* < 0.0001. In post hoc testing for Šídák’s multiple comparisons, ASC was different between each individual IVH patient compared with the controls, **** *p* < 0.0001 (Figure 2A). Moreover, caspase-1 concentration levels, analyzed with a two-way ANOVA, were significantly higher in the CSF of IVH patients compared to controls *F*(5, 31) = 7.060, **** *p* < 0.0002. In post hoc testing for Šídák’s multiple comparisons, caspase-1 was different between IVH patient 2 vs. controls (n = 3), * *p* < 0.0124; IVH patient 3 vs. controls (n = 3), **** *p* < 0.0001; IVH patient 5 vs. controls (n = 3), * *p* < 0.0145; and not significant (ns) between IVH patient 1 vs. controls (n = 3), *p* < 0.1057 and between IVH patient 4 vs. controls (n = 3), *p* < 0.9658 (Figure 2B). Lastly, IL-18 concentration levels were significantly higher in the CSF of IVH patients compared with the controls, *F*(5, 31) = 30.15, **** *p* < 0.0001, as assessed using a two-way ANOVA. In post hoc testing for Šídák’s multiple comparisons, IL-18 was different between IVH patients 1, 2, 3, 4 vs. controls (n = 3), **** *p* < 0.0001, and between IVH patient 5 vs. controls (n = 3), *** *p* < 0.0004 (Figure 2C). Given that the controls (n = 3) each had a single collection time point, these three samples are represented separately from the CTPs.

**Figure 3:**
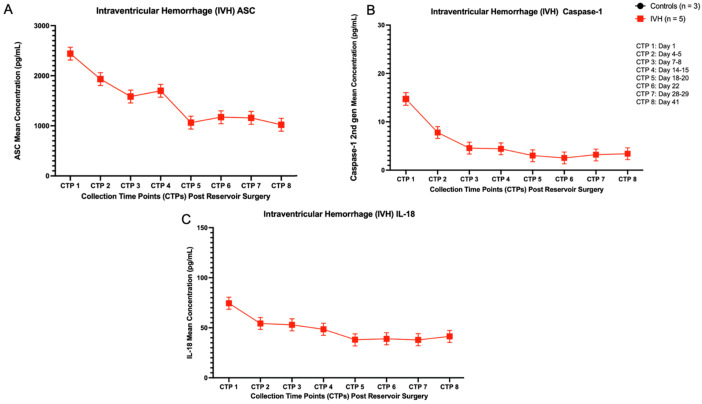
Averaged inflammasome protein concentrations in IVH patients (n = 5) at each CSF collection time point. The paired t-test results for the inflammasome protein concentration levels were significant: (**A**) ASC—*p* < 0.0001, *F*(7) = 7.564; (**B**) *p* < 0.0072, *F*(7) = 3.745; (**C**) *p* < 0.0001, *F*(7) = 10.13. Given that these controls (n = 3) each have a single collection time point, these values are not reflected in the graphs. Controls (n = 3) mean concentrations: ASC (167 pg/mL), caspase-1 (0.039 pg/mL), IL-18 (3.57 pg/mL).

Subsequently, at each CSF collection time point, the inflammasome concentration levels (ASC, caspase-1, IL-18) for the IVH patients (n = 5) were averaged and compared to the average of the healthy controls (n = 3). The paired t-test results for ASC concentration levels were significant, **** *p* < 0.0001, *F*(7) = 7.564 (Figure 3A). The paired t-test results for caspase-1 concentration levels were significant, ** *p* < 0.0072, *F*(7) = 3.745 (Figure 3B). Lastly, the paired t-test results for IL-18 concentration levels were significant, **** *p* < 0.0001, *F*(7) = 10.13 (Figure 3C). Inflammasome protein concentrations were highest on day 1 post-reservoir surgery, with variable shifts, including a subsequent concentration elevation, particularly in ASC and caspase-1, approximately two weeks post-reservoir surgery. Given that these controls (n = 3) each had a single collection time point, these values are not reflected in the graphs. Controls (n = 3) mean concentrations: ASC (167 pg/mL), caspase-1 (0.039 pg/mL), IL-18 (3.57 pg/mL).

### 3.3. Preliminary ROC Analysis Identifies ASC, Caspase-1, and IL-18 as Promising Biomarkers for Pediatric Hydrocephalus Compared to Healthy Controls

**Figure 4:**
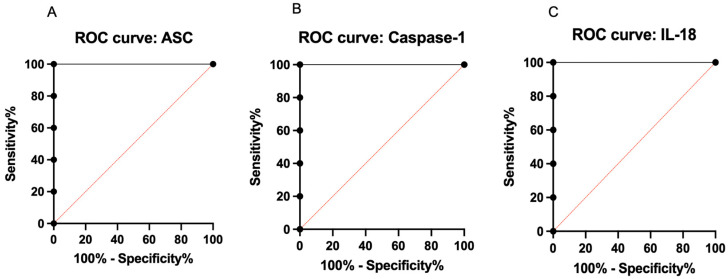
ROC curves for inflammasome proteins at CTP 1 ((**A**)—ASC, (**B**)—caspase-1, and (**C**)—IL-18) were fitted to compare IVH patients (n = 5) vs. healthy controls (n = 3) and indicate the AUC (sensitivity % vs. 1-specificity %) for statistically significant biomarker levels.

Two ROC analyses were performed. The first ROC analysis was performed at CTP 1 for ASC, caspase-1, and IL-18, given that this was the most acute time-point post-reservoir surgery to detect discriminatory performance between IVH patients and controls. The datasets for ASC, caspase-1, and IL-18 were then used to generate the ROC metrics (cut-off, sensitivity, specificity, AUC) (Table S1). The ROC metrics were subsequently fitted in ROC curves (Figure 4). The analysis revealed promising findings with a sensitivity of 100%, specificity of 100%, an AUC of 1.0, a positive predictive value (PPV) of 100%, and a negative predictive value (NPV) of 100% at their corresponding cut-off points. The 95% confidence interval for each inflammasome protein ranged from 56.55% to 100.0%, with ** *p* < 0.0090 illustrating statistical significance (Table S1).

In the second ROC analysis, for each inflammasome protein (ASC, caspase-1, and IL-18), the ROC data were generated separately at each of the eight collection timepoints (CTPs). At each CTP, the dataset included five values for the IVH patients and three values for the healthy controls. The ROC metrics (cut-off, sensitivity, specificity, and AUC) were calculated to assess the discriminatory performance between the IVH patients and controls. A sensitivity of 80%, a specificity of 100%, an AUC of 1.0, and an SE of 0.0 indicate high discriminatory ability between pediatric hydrocephalic patients versus controls at their corresponding cut-off points. The CI for the AUC ranged from 51.01% to 100% or from 56.55% to 100%, and the *p*-values were statistically significant (* *p* < 0.0209 or ** *p* < 0.0090), supporting the potential reliability of these biomarkers (Table S2).

## 4. Discussion

To our knowledge, this study is the first to analyze inflammasome protein concentration levels in the CSF of pediatric patients with hydrocephalus due to IVH of prematurity post-reservoir surgery, compared to healthy controls. First, we demonstrated that, despite having an immature immune system, inflammasome proteins are involved in pediatric hydrocephalus, since the protein concentrations are both detectable and elevated in the CSF of IVH patients versus healthy controls. Second, our study is innovative and potentially beneficial for the field of neonatology and pediatric neurosurgery, since we identified ASC, caspase-1, and IL-18 as promising and measurable biomarkers for pediatric hydrocephalus. Third, this study provides insights into future studies that can be conducted to evaluate the diagnostic and prognostic ability of these inflammasome proteins, including predicting the appropriate interval for further clinical and surgical intervention.

Our findings indicate that there is an association between the presence of inflammasome proteins in the CSF of patients with hydrocephalus due to IVH of prematurity. In IVH, there is blood deposition in the cerebral ventricles and cisterna magna, which can result in mechanical damage to neighboring tissues, impairment of resorption, or scarring blocking outflow [36,37]. Plasma components and subsequent erythrocyte lysis contribute to the release of hemoglobin and its oxidized forms that produce radical oxygen species (ROS) [36]. These oxidized metabolites target cells, including glial cells, choroid plexus epithelial cells, and brain microvascular endothelial cells, a key component of the blood–brain barrier (BBB) [38,39,40]. These previously reported mechanisms in IVH contribute to an elevation of ASC, caspase-1, and IL-18.

There is a need to investigate the inflammasome protein expression levels in pediatric hydrocephalus. Our findings indicate that inflammasome proteins are elevated in pediatric hydrocephalus due to IVH of prematurity. ASC is directly activated by NLRP3 (nucleotide-binding domain, leucine-rich repeat, and pyrin domain-containing protein 3) [41,42]. Although no currently published literature has directly studied ASC levels in pediatric hydrocephalus, researchers have investigated NLRP3 activation in hydrocephalus. Zhang and colleagues revealed that NLRP3 levels were elevated in cells of the choroid plexus in a rat hydrocephalus model, and that subsequent knockout of NLRP3 ameliorated hydrocephalic symptoms [43,44]. Peng and colleagues obtained serum samples from adult patients who developed hydrocephalus post-operatively and found the NLRP3 levels to be elevated via ELISA analysis [45]. Furthermore, elevated caspase-1 levels have been implicated in various neuroinflammatory conditions. For instance, research studies involving animal models and adult patients with subarachnoid hemorrhage (SAH) or acute hydrocephalus have reported increased caspase-1 expression in both pathologies [46,47]. Research implementing ELISA methodology by Schmitz and Sival has also demonstrated elevated IL-18 levels in the CSF of preterm infants with hydrocephalus compared to controls [48,49].

Our study is novel in that it identifies ASC, caspase-1, and IL-18 as measurable and promising biomarkers for pediatric hydrocephalus. Additionally, the diagnostic performance of inflammasome proteins has been validated in other inflammatory conditions using ROC curves and AUC analyses. These conditions include TBI [24,25,50,51,52], Alzheimer’s disease [27,53,54], SAH [28,47,55,56], and meningitis [57,58,59]. Our study does have limitations. These include a small sample size, slight variations in CSF sample CTPs, and patient cohorts taken from a single hospital setting. It is important to acknowledge, however, the unique sample size challenges faced when conducting research in the NICU patient population. The difficulties include the fact that these patients are a vulnerable population that does not have the capacity or ability to give informed consent; rather, this is obtained from their families, who may or may not be willing to participate in clinical studies, given the delicate condition of their child [60,61,62,63]. Additionally, hydrocephalus in the pediatric population is relatively uncommon compared to other adult pathologies, thus making it more challenging to have a large sample size compared to adult studies [64]. Our present study is focused on hydrocephalus due to IVH and does not include other etiologies. Therefore, these findings cannot be generalized to all causes of hydrocephalus. Furthermore, since CSF was collected for clinical, rather than research purposes, we attempted to match CTPs as closely as possible, but they were not performed at exactly the same timepoint across all patients. We included multiple time points for each patient, although samples were collected from one center. Despite these limitations, the CSF sample analysis produced promising ROC and AUC results. Our ROC curve analysis produced an AUC of 1.0, a positive predictive value (PPV) of 100%, and a negative predictive value (NPV) of 100% for ASC, caspase-1, and IL-18 with a high sensitivity and specificity. The similar statistical results may, in part, be attributed to the close functional linkage of these inflammasome proteins within the inflammasome cascade. Consequently, the ROC analysis distinguished between the pathological hydrocephalic and healthy states.

Currently, the primary mechanisms to treat hydrocephalus involve surgical intervention. This research has provided insights into future research directions, particularly the potential prognostic ability of these inflammasome proteins to predict the appropriate interval for further clinical and surgical intervention. In our averaged analysis of IVH patients versus healthy controls, day 1 post-reservoir surgery exhibited the highest concentration level. The second highest concentration levels occurred at approximately two weeks post-reservoir surgery for ASC and caspase-1. Previous studies have indicated that the inflammasome complex involves a biphasic immune activation, including activation and priming [65,66,67]. The second phase may be delayed in conditions such as IVH, SAH, and intracranial hemorrhage (ICH) [68,69,70]. This study is longitudinal, and enrollment is ongoing. Given our limited sample size, this feasibility study presents promising preliminary data to demonstrate the efficacy of pursuing further CSF biomarkers studies in a larger cohort of neonatal patients. The enrollment of additional patients will be based on clinical presentation and surgical treatment at Jackson Memorial Hospital. This increase in sample size will enable us to elucidate the inflammatory trends further, providing insights that could be diagnostic and prognostic for clinical interventions. In our cohort, four of the five patients required permanent CSF diversion. The baby who did not receive a VP shunt was more likely clinically unstable compared to the other participants. A future goal is to determine whether patients who undergo reservoir placement but do not later require permanent CSF diversion may have differences in inflammasome protein levels. Future studies will also involve larger cohorts of pediatric hydrocephalic patients due to varying etiologies (i.e., meningitis, spina bifida, IVH) to enhance the generalizability of our findings, more collection time points, and the investigation of other inflammasome protein concentration levels post-reservoir surgery. Understanding the pathophysiology of the inflammasome complex in pediatric hydrocephalus may also lead to potential pharmacological and therapeutic targets.

## 5. Conclusions

Our study demonstrated that in pediatric hydrocephalus due to IVH of prematurity, ASC, caspase-1, and IL-18 concentration levels were detectable and elevated compared to healthy controls. Preliminary ROC and AUC analysis data indicated that these inflammasome proteins are potential biomarkers for pediatric hydrocephalus. Future studies involving pediatric hydrocephalic patients may include larger sample sizes, more sample collection time points, additional etiologies, and investigation of other inflammasome protein concentrations in the CSF. Our findings reveal inflammasome proteins as potential biomarkers for pediatric hydrocephalus; however, future research strives to elucidate inflammatory trends to enhance the appropriate window of surgical intervention and ultimately improve clinical outcomes.

## Data Availability

The raw data used for ROC curve analysis is available in the supplemental table (Table S1).

## Supplementary Materials

The following supporting information can be downloaded at https://www.mdpi.com/article/10.3390/biom16010035/s1. Table S1: ROC analysis for ASC, caspase-1, and IL-18 for collection time point 1 (CTP 1); Table S2: ROC analysis for ASC, caspase-1, and IL-18 at each CSF collection time point (CTP).

## Abbreviations

The following abbreviations are used in this manuscript:

CSF	cerebrospinal fluid
IVH	intraventricular hemorrhage
VAD	ventricular access device
VSGS	ventriculosubgaleal shunt
VP shunt	ventriculoperitoneal shunt
ETV	endoscopic third ventriculostomy
ASC	apoptosis associated speck-like protein containing a caspase recruitment domain
IL-18	interleukin-18
AUC	area under the curve
ROC	receiver operator characteristics
NICU	neonatal intensive care unit
ANOVA	analysis of variance
SE	standard error
CI	confidence interval
TBI	traumatic brain injury
SAH	sub-arachnoid hemorrhage
ICH	intracranial hemorrhage
