# Supplementary material for: Feasibility Study on Inflammasome Proteins as Biomarkers in the Cerebrospinal Fluid of Pediatric Patients with Hydrocephalus Due to Intraventricular Hemorrhage
Source: Biomolecules. 2025 Dec 25;16(1):35. doi: 10.3390/biom16010035 (PMC12838929; doi:10.3390/biom16010035)
Supplement: Supplementary file 1 [file biomolecules-16-00035-s001.zip › biomolecules-4026690-supplementary.pdf]

Table S1. ROC Analysis for ASC, caspase-1, IL-18 for CTP 1: For the corresponding cutoff points (pg/mL), sensitivity (100%), specificity (100%), area under the curve (AUC), standard error (SE), 95% confidence interval (CI).

Inflammasome proteins	Cutoff points (pg/mL)	Sensitivity	Specificity	AUC	SE	95% CI	*p*-value
ASC							
	>847.0	100%	100%	1.0	0.0	56.55% to 100%	** $p < 0.0090$
	>1824	80%	100%	1.0	0.0	56.55% to 100%	** $p < 0.0090$
	>2441	60%	100%	1.0	0.0	56.55% to 100%	** $p < 0.0090$
	>2789	40%	100%	1.0	0.0	56.55% to 100%	** $p < 0.0090$
	>2900	20%	100%	1.0	0.0	56.55% to 100%	** $p < 0.0090$
Caspase-1							
	>0.6995	100%	100%	1.0	0.0	56.55% to 100%	** $p < 0.0090$
	>5.300	80%	100%	1.0	0.0	37.55% to 98.97%	** $p < 0.0090$
	>12.52	60%	100%	1.0	0.0	23.07% to 92.89%	** $p < 0.0090$
	>18.50	40%	100%	1.0	0.0	7.107% to 76.93%	** $p < 0.0090$
	>23.60	20%	100%	1.0	0.0	1.026% to 62.45%	** $p < 0.0090$
IL-18							
	>26.99	100%	100%	1.0	0.0	56.55% to 100%	** $p < 0.0090$
	>52.90	80%	100%	1.0	0.0	56.55% to 100%	** $p < 0.0090$
	>56.45	60%	100%	1.0	0.0	56.55% to 100%	** $p < 0.0090$
	>76.85	40%	100%	1.0	0.0	56.55% to 100%	** $p < 0.0090$
	>104.6	20%	100%	1.0	0.0	56.55% to 100%	** $p < 0.0090$

Table S2. ROC Analysis for ASC, caspase-1, IL-18 at each CSF collection time point (CTP): For the corresponding cutoff points (pg/mL), sensitivity (80%), specificity (100%), area under the curve (AUC), standard error (SE), 95% confidence interval (CI).

Inflammasome proteins	Cutoff points (pg/mL)	Sensitivity	Specificity	AUC	SE	95% CI	*p*-value
ASC							
CTP 1	>1824	80%	100%	1.0	0.0	56.55% to 100%	** $p < 0.0090$
CTP 2	>1259	80%	100%	1.0	0.0	56.55% to 100%	** $p < 0.0090$
CTP 3	>1218	80%	100%	1.0	0.0	56.55% to 100%	** $p < 0.0090$
CTP 4	>1495	75%	100%	1.0	0.0	51.01% to 100%	* $p < 0.0209$
CTP 5	>766.5	80%	100%	1.0	0.0	56.55% to 100%	** $p < 0.0090$
CTP 6	>852	75%	100%	1.0	0.0	51.01% to 100%	* $p < 0.0209$
CTP 7	>699	75%	100%	1.0	0.0	51.01% to 100%	* $p < 0.0209$
CTP 8	>823	75%	100%	1.0	0.0	51.01% to 100%	* $p < 0.0209$
Caspase-1							
CTP 1	>5.3	80%	100%	1.0	0.0	56.55% to 100%	** $p < 0.0090$
CTP 2	>2.3	80%	100%	1.0	0.0	56.55% to 100%	** $p < 0.0090$
CTP 3	>2.43	80%	100%	1.0	0.0	56.55% to 100%	** $p < 0.0090$
CTP 4	>2.49	75%	100%	1.0	0.0	51.01% to 100%	* $p < 0.0209$
CTP 5	>1.226	80%	100%	1.0	0.0	56.55% to 100%	** $p < 0.0090$
CTP 6	>1.588	75%	100%	1.0	0.0	51.01% to 100%	* $p < 0.0209$
CTP 7	>1.574	75%	100%	1.0	0.0	51.01% to 100%	* $p < 0.0209$
CTP 8	>2.123	75%	100%	1.0	0.0	51.01% to 100%	* $p < 0.0209$
IL-18							
CTP 1	>52.90	80%	100%	1.0	0.0	56.55% to 100%	** $p < 0.0090$
CTP 2	>40.65	80%	100%	1.0	0.0	56.55% to 100%	** $p < 0.0090$
CTP 3	>42.35	80%	100%	1.0	0.0	56.55% to 100%	** $p < 0.0090$
CTP 4	>40.15	75%	100%	1.0	0.0	51.01% to 100%	* $p < 0.0209$
CTP 5	>21.95	80%	100%	1.0	0.0	56.55% to 100%	** $p < 0.0090$
CTP 6	>25	75%	100%	1.0	0.0	51.01% to 100%	* $p < 0.0209$
CTP 7	>29.85	75%	100%	1.0	0.0	51.01% to 100%	* $p < 0.0209$
CTP 8	>21.60	75%	100%	1.0	0.0	51.01% to 100%	* $p < 0.0209$
